# Heritability Estimates of Behavioral Traits and Their Genetic Relationships with Performance Traits in Japanese Quail

**DOI:** 10.3390/ani16131943

**Published:** 2026-06-23

**Authors:** Esra Karaduman, Doğan Narinç, Ali Aygun

**Affiliations:** 1 International Center for Livestock Research and Training, 06852 Ankara, Turkey; esra.karaduman@tarimorman.gov.tr; 2 Department of Animal Science, Faculty of Agriculture, Akdeniz University, 07070 Antalya, Turkey; 3 Department of Animal Science, Faculty of Agriculture, Selçuk University, 42130 Konya, Turkey; aaygun@selcuk.edu.tr

**Keywords:** behavioral traits, animal welfare, heritability, genetic correlations, multi-trait animal model

## Abstract

The present study aimed to estimate the heritabilities of behavioral, growth, carcass, and growth curve traits and to determine the genetic and phenotypic relationships among these traits in Japanese quail (*Coturnix japonica*). A total of 500 quail chicks originating from a pedigree population were evaluated for growth performance, carcass characteristics, growth curve parameters, and behavioral traits. Genetic parameters were estimated using multi-trait animal models based on pedigree information. Heritability estimates for body weight traits ranged from 0.19 to 0.71, indicating substantial additive genetic variation for growth performance. Feed conversion ratio traits showed low to moderate heritability estimates (0.06–0.45), whereas growth curve parameters exhibited heritabilities ranging from 0.13 to 0.44. Among behavioral traits, feather pecking (0.35), wing stretching (0.30), and aggressive pecking (0.28) displayed the highest heritability estimates. Favorable genetic and phenotypic relationships were observed between feeding and inactivity behaviors and growth and carcass traits, while walking behavior showed moderate negative genetic associations with BW42. In contrast, welfare-related behavioral traits generally exhibited weak relationships with production traits. Genetic correlations among several behavioral traits suggested the existence of common biological mechanisms influencing behavioral expression. These findings demonstrated that behavioral traits possess exploitable genetic variation and should be considered alongside conventional production traits in future poultry breeding programs aimed at improving both productivity and animal welfare.

## Simple Summary

Behavioral traits in Japanese quail showed measurable genetic variation and meaningful relationships with growth and carcass traits. Particularly, feather pecking and aggressive pecking exhibited relatively higher heritability estimates. The findings suggest that selected behavioral traits may provide additional information for future poultry breeding programs aiming to balance productivity and animal welfare.

## 1. Introduction

The intensification of poultry production systems over recent decades has substantially improved growth performance, feed efficiency, and carcass yield through continuous genetic selection and advances in management practices. However, the strong emphasis placed on production efficiency has also raised important concerns regarding animal welfare, behavioral adaptability, and physiological resilience in modern poultry populations. Rapid genetic progress for economically important production traits has, in some cases, been accompanied by increased susceptibility to stress, behavioral disorders, impaired locomotor activity, and reduced adaptability to environmental challenges [1,2]. Consequently, sustainable poultry production systems increasingly require breeding strategies that simultaneously consider productivity, animal welfare, and biological robustness.

Animal welfare is now recognized as a multidimensional concept encompassing not only health and productivity, but also the ability of animals to express natural behaviors and adapt successfully to their environment [3,4]. In poultry species, behavioral traits are considered among the most informative welfare indicators because behavioral responses directly reflect the interaction between the animal and its physical and social environment [5]. Behaviors such as feeding, locomotion, resting, preening, feather pecking, and aggressive interactions provide valuable information regarding stress responsiveness, social hierarchy, environmental adaptation, and overall welfare status. Therefore, behavioral phenotypes have gained increasing importance in both welfare assessment protocols and modern poultry breeding programs.

Although behavioral traits are strongly influenced by environmental conditions, a substantial body of evidence indicates that many poultry behaviors also possess a measurable genetic basis [6]. Previous studies conducted mainly in laying hens and broiler chickens demonstrated that behaviors associated with feather pecking, aggression, social interactions, fear responses, and activity patterns exhibit low to moderate heritability estimates and may show important genetic correlations with production traits [7,8,9]. These findings suggest that behavioral traits are not merely environmentally induced responses, but complex phenotypes shaped through interactions between genetic background and environmental conditions. Consequently, behavioral characteristics may potentially be incorporated into selection programs aimed at improving both animal welfare and production sustainability.

Traditional poultry breeding programs have primarily focused on economically important traits such as body weight, body weight gain, feed conversion ratio, and carcass yield. However, exclusive selection for rapid growth and high production efficiency may unintentionally increase the prevalence of welfare-related problems, including reduced locomotor activity, skeletal disorders, feather pecking, and social stress [1,10]. For this reason, increasing attention has recently been directed toward welfare-oriented breeding approaches integrating behavioral traits into multi-trait genetic evaluation systems [11,12]. The estimation of heritabilities and genetic correlations between behavioral and production traits is therefore essential for understanding whether welfare-related behaviors can be improved without generating major antagonistic effects on economically important production characteristics.

Japanese quail (*Coturnix japonica*) represents an important avian model for genetic, physiological, nutritional, and behavioral studies due to its short generation interval, rapid growth rate, early sexual maturity, high reproductive capacity, and ease of management under controlled experimental conditions [13,14]. In addition to its economic importance in poultry production, the species is widely used as a biological model for evaluating growth biology, stress physiology, reproductive performance, and behavioral responses. Despite the increasing interest in poultry welfare and behavioral genetics, studies simultaneously evaluating behavioral traits, production characteristics, carcass traits, and growth curve parameters within a multi-trait genetic framework remain limited in Japanese quail populations. Most previous studies conducted in quail have primarily focused on growth performance, feed efficiency, carcass characteristics, or nonlinear growth modeling separately [15,16,17]. In contrast, information regarding the genetic architecture of behavioral traits and their relationships with production-related characteristics remains scarce. Particularly, the extent to which behavioral phenotypes are genetically associated with growth performance, feed efficiency, and carcass yield has not been comprehensively clarified in Japanese quail populations. Understanding these relationships may provide valuable insights into the development of balanced breeding strategies integrating both productivity and animal welfare. Therefore, the present study aimed to estimate the heritabilities of behavioral, growth, carcass, and growth curve traits in Japanese quail and to determine the genetic and phenotypic relationships among these traits using multi-trait animal models based on pedigree information. It was hypothesized that behavioral traits possess measurable additive genetic variation and exhibit biologically meaningful relationships with production-related traits. The findings obtained from the present study may contribute to the development of sustainable and welfare-oriented poultry breeding strategies integrating behavioral phenotypes together with conventional production traits.

## 2. Materials and Methods

### 2.1. Experimental Animals and Management

All experimental procedures involving animals were conducted in accordance with national and institutional guidelines for the care and use of experimental animals. The animal material of the present study consisted of 500 Japanese quail (*Coturnix japonica*) chicks obtained from a non-selected base population comprising 40 males and 120 females maintained at the Poultry Research Unit of the Department of Animal Science, Faculty of Agriculture, Akdeniz University, Antalya, Türkiye. Fertile eggs were collected from the breeding flock, incubated under standard commercial incubation conditions, and chicks were individually identified using wing tags immediately after hatch to establish pedigree records and maintain individual identification throughout the experimental period.

Birds were reared from hatch to 42 days of age under standardized environmental and management conditions. During the brooding period, ambient temperature was maintained at 36 °C during the first week and gradually reduced by approximately 3 °C per week until reaching 24 °C. A continuous lighting program (23L:1D) was applied during the first week, after which a 16L:8D lighting schedule was maintained until the end of the experiment. Relative humidity was maintained between 55% and 65% throughout the experimental period.

Quails were housed in a total of fourteen floor pens under controlled environmental conditions at a stocking density of 384 cm^2^ per bird. Pens were equipped with deep litter consisting of coarse wood shavings, with an initial litter depth of approximately 3 cm at placement. Feed and water were provided ad libitum throughout the experimental period. Birds were fed a commercial grower diet formulated for Japanese quail containing approximately 23% crude protein and 2900 kcal/kg metabolizable energy. The diet was balanced for essential amino acids as well as calcium and available phosphorus requirements. The commercial diet was formulated according to NRC recommendations for growing Japanese quail [18]. Health status and mortality were monitored daily.

### 2.2. Data Collection and Preparation

Individual body weights were recorded weekly from hatch to 42 days of age using a digital precision scale with 0.01 g sensitivity. In the present study, body weight traits recorded at 35 and 42 days of age (BW35 and BW42) were included in the genetic analyses. Feed intake was recorded on a pen basis at weekly intervals. Cumulative feed conversion ratio (FCR) values for 35 and 42 days of age were calculated using pen-level cumulative feed intake and individual body weight gain during the corresponding periods according to the following equation:

$$FCR=\frac{Feed\;Intake}{Body\;Weight\;Gain}$$

Because feed intake measurements were obtained at the pen level, the calculated FCR values should be interpreted as individual gain-adjusted feed conversion indicators rather than direct individual feed intake-based FCR measurements.

Prior to estimating individual growth curve parameters, five nonlinear growth models commonly used in poultry growth studies, namely Logistic, Richards, Morgan–Mercer–Flodin (MMF), Gompertz, and von Bertalanffy functions, were fitted to the body weight data. Model comparison was based on Akaike Information Criterion (AIC), Bayesian Information Criterion (BIC), and coefficient of determination (R^2^). The goodness-of-fit statistics for the evaluated nonlinear growth models are presented in Table 1. The Logistic model represents a symmetric sigmoidal growth pattern, whereas the Richards model includes an additional shape parameter allowing greater flexibility in curve asymmetry. The MMF model combines sigmoidal and asymptotic growth characteristics, while the von Bertalanffy model has been widely applied to describe biological growth processes in poultry and other livestock species. Among the evaluated models, the Gompertz function provided the best overall fit according to all goodness-of-fit criteria and was therefore selected for subsequent estimation of individual growth curve parameters.

Growth curve characteristics were estimated using the three-parameter Gompertz nonlinear growth model:

$$W_{t}=\beta_{0}exp(-\beta_{1}e^{-\beta_{2}t})$$

where $W_{t}$ represents body weight at age t, $\beta_{0}\;$ is the asymptotic mature weight, $\beta_{1}\;$ is the integration constant, and $\beta_{2\;}$ represents the growth rate parameter. Based on Gompertz model parameters, age at the inflection point (IPA) and body weight at the inflection point (IPW) were calculated for each individual. Nonlinear growth analyses were performed using individual body weight records obtained during the experimental period.

Behavior was evaluated during the fifth and sixth weeks of age using the focal animal sampling method [19]. Prior to data collection, birds were allowed a short adaptation period to minimize disturbance-related responses. Each focal session lasted 300 s per individual under standardized environmental conditions. Assessments were distributed across consecutive days using a rotational sampling design to ensure repeated measurements for all birds. Observations were conducted during scheduled morning and afternoon periods (10:00 and 14:00 h) by three trained observers following standardized behavioral definitions. Data were recorded in real time using The Observer XT software version 17 (Noldus Information Technology, Wageningen, The Netherlands) and subsequently verified from video footage. Inter-observer reliability was assessed using intraclass correlation coefficients (ICC) calculated across the three observers. ICC values ranged from 0.85 to 0.98, indicating good to excellent observer agreement according to Koo and Li [20]. Each individual was evaluated repeatedly during both the fifth and sixth weeks of age.

The behavioral repertoire evaluated in the present study included performance-related activity behaviors and welfare-related behaviors. Performance-related activity behaviors consisted of feeding, drinking, walking, inactivity, and scratching, whereas welfare-related behaviors included maintenance and comfort behaviors, such as wing stretching, shaking, and preening, as well as socially directed behaviors, such as feather pecking and aggressive pecking. Behavioral definitions were adapted from established poultry ethograms reported in previous behavioral studies [21,22,23,24,25,26,27,28].

Behavioral traits were defined as follows:

Feeding: Consumption of feed from the feeder.
Drinking: Consumption of water from the water source (nipple).
Walking: Locomotion activity within the pen.
Inactivity: Inactive sitting, resting, or standing posture without locomotion.
Scratching: Scratching or ground-pecking activity.
Wing stretching: Unilateral or bilateral wing extension movements.
Shaking: Rapid body or feather shaking movements.
Preening: Feather grooming and maintenance behavior.
Feather pecking: Pecking directed toward feathers of another bird.
Aggressive pecking: Forceful pecking directed toward another bird accompanied by aggressive interactions.

Behavioral measurements were recorded as direct duration-based observational variables within standardized observation periods. Direct behavioral durations were preferred over proportional time-budget measures in order to retain individual-level phenotypic variability for genetic analyses.

At 42 days of age, all birds included in the experimental population were slaughtered following standard commercial poultry slaughter procedures. Prior to slaughter, birds were fasted for approximately 6 h while maintaining free access to water. After slaughter and evisceration, carcass traits including cold carcass weight, breast weight, thigh weight, wing weight, abdominal fat weight, and edible internal organ weights were recorded using a digital precision scale according to standard poultry carcass evaluation procedures described for Japanese quail [29]. Carcass percentages were calculated relative to cold carcass weight.

### 2.3. Statistical and Genetic Analyses

Descriptive statistics for all evaluated traits were calculated prior to genetic analyses. Prior to genetic analyses, trait distributions were examined for normality and homogeneity of variance. Body weight, feed conversion ratio, and carcass traits were analyzed using their original measurements. Behavioral traits were subjected to square-root transformation [√(x + 1)] to reduce skewness associated with low-frequency and zero-inflated observations. Growth curve parameters exhibiting departures from normality were transformed using the Box–Cox procedure, with trait-specific transformation parameters of λ = −1 for β1, λ = 2 for β2, λ = −1.1 for IPA, and λ = −2 for IPW. Fluctuating asymmetry traits were standardized using z-score transformation prior to the calculation of composite asymmetry indices. Heritability estimates as well as genetic and phenotypic correlations among behavioral, growth, carcass, and growth curve traits were estimated using pedigree-based multi-trait animal models. Prior to analyses, residual distributions and model assumptions were assessed graphically. Traits exhibiting non-Gaussian distributions were normalized using logarithmic (ln) or Box–Cox transformations where appropriate prior to REML analyses.

The pedigree dataset consisted of 1347 animals traced across three generations. Additive genetic relationship matrices were constructed using pedigree information obtained from the experimental population. Multi-trait pedigree-based animal models were fitted using the sommer package in R, which allows for the estimation of variance–covariance structures under restricted maximum likelihood (REML) procedures [30]. Multi-trait animal models were fitted for trait combinations of interest to estimate variance–covariance components as well as genetic and phenotypic correlations among behavioral, growth, carcass, and growth curve traits. The general linear mixed animal model used for genetic analyses was as follows:

y=Xb+Za+e

where y is the vector of observations, b is the vector of fixed effects, a is the vector of random additive genetic effects, and e is the vector of random residual effects. X and Z represent the corresponding incidence matrices relating observations to fixed and random effects, respectively.

Sex was included as a fixed effect in all models, and the corresponding sex-effect statistics for the evaluated traits are summarized in Supplementary Table S1. Birds were hatched within a relatively narrow time window and reared under highly standardized environmental and management conditions. Therefore, hatch and pen effects were not retained in the final models to maintain model parsimony and computational stability during multi-trait REML analyses. Nevertheless, potential residual common environmental influences associated with shared housing conditions should be considered when interpreting the estimated genetic parameters, particularly for behavioral traits.

The covariance structures for additive genetic and residual effects were assumed as:

$$Var\left(a\right)=A⊗G$$

$$Var\left(e\right)=I⊗R$$

where A is the numerator relationship matrix derived from pedigree information, G is the additive genetic covariance matrix among traits, I is the identity matrix, and R is the residual covariance matrix.

Heritability estimates were calculated as:

$$h^{2}=\frac{\sigma_{a}^{2}}{\sigma_{p}^{2}}$$

where $\sigma_{a}^{2}$ represents additive genetic variance and $\sigma_{p}^{2}$ represents total phenotypic variance. Genetic correlations are presented together with their corresponding standard errors (SE), whereas phenotypic correlations are presented together with their associated probability levels (*p*-values). Genetic correlations were obtained from the estimated additive genetic covariance matrices generated by the multi-trait animal models, while phenotypic correlations were calculated using Pearson correlation coefficients based on individual phenotypic records.

Model convergence was evaluated by monitoring the change in restricted log-likelihood values across iterations and by examining the positive definiteness of the estimated variance–covariance matrices. All fitted multi-trait models converged successfully and satisfied the convergence criteria implemented in the sommer package of R statistical software version 4.5.2 (R Foundation for Statistical Computing, Vienna, Austria).

Individual growth curve parameters were estimated using the three-parameter Gompertz nonlinear growth model fitted to individual body weight records using nonlinear least squares regression procedures implemented in the nlme package of R statistical software version 4.5.2 (R Foundation for Statistical Computing, Vienna, Austria) [30,31].

## 3. Results

Descriptive statistics and heritability estimates for growth performance traits are presented in Table 2. Body weight traits exhibited higher heritability estimates than feed efficiency traits (Table 2). BW35 and BW42 showed moderate-to-high additive genetic determination (h^2^ = 0.55 and 0.53, respectively), whereas FCR traits exhibited moderate heritability estimates. Phenotypic variability was generally moderate across growth performance traits, with CV values ranging between 9.09% and 12.12%.

Table 3 presents the descriptive statistics and heritability estimates for Gompertz growth curve parameters in Japanese quail. Moderate heritability estimates were obtained for all Gompertz growth curve parameters (Table 3). β_0_ and IPW exhibited the greatest phenotypic variability, whereas β_1_ showed lower variation. Individual Gompertz analyses showed excellent goodness-of-fit (R^2^ = 0.9892–0.9999), confirming the suitability of the nonlinear model for describing quail growth trajectories.

Descriptive statistics for slaughter and carcass traits are shown in Table 4. Cold carcass and breast weights exhibited comparatively higher heritability estimates than abdominal fat and wing traits (Table 4). Abdominal fat showed substantially greater phenotypic variability than the remaining carcass traits.

Descriptive statistics and heritability estimates for behavioral traits are presented in Table 5. Behavioral traits exhibited substantially greater phenotypic variability than production traits (Table 5). Feather pecking, wing stretching, and aggressive pecking showed the highest heritability estimates among behavioral traits, whereas preening and inactivity exhibited weaker additive genetic determination.

Table 6 presents the genetic (r_G_) and phenotypic (r_P_) correlations between performance-related activity behaviors and production traits in Japanese quail. Feeding behavior exhibited moderate positive correlations with BW42 at both genetic and phenotypic levels (r_G_ = 0.45 ± 0.01; r_P_ = 0.36, *p* < 0.001), whereas negative correlations were detected with FCR42 and IPA. Similarly, inactivity behavior showed positive associations with BW42 and cold carcass weight, but negative correlations with FCR42 and IPA. Walking behavior exhibited negative correlations with BW42 at both genetic and phenotypic levels (r_G_ = −0.38 ± 0.02; r_P_ = −0.33, *p* < 0.001). In contrast, correlations between walking behavior and FCR42 were close to zero. Weak positive genetic correlations were observed between walking behavior and both IPA and cold carcass weight. Most correlations involving drinking and scratching behaviors were low and statistically non-significant at both genetic and phenotypic levels. The stronger associations with production traits were observed for feeding, walking, and inactivity behaviors compared with drinking and scratching behaviors.

Table 7 summarizes the genetic (r_G_) and phenotypic (r_P_) correlations between welfare-related behavioral traits and production traits in Japanese quail. In general, welfare-related behavioral traits exhibited weak correlations with the evaluated production traits at both genetic and phenotypic levels, and most phenotypic correlations were statistically non-significant (*p* > 0.05). Wing stretching showed weak positive correlations with BW42 and cold carcass weight, whereas weak negative correlations were observed with FCR42 and IPA. Similarly, preening behavior exhibited generally low correlations with production traits, with slight negative associations with IPA at both genetic and phenotypic levels. Among the evaluated social behaviors, shaking behavior showed negative correlations with BW42, IPA, and cold carcass weight, whereas positive correlations were observed with FCR42. Aggressive pecking exhibited near-zero correlations with BW42 and cold carcass weight, while weak positive correlations were detected with FCR42.

## 4. Discussion

Body weight traits exhibited moderate-to-high heritability estimates, confirming that growth performance in Japanese quail is strongly influenced by additive genetic effects. Similar heritability ranges for body weight traits have consistently been reported in quail populations by Minvielle [13], Narinç et al. [15], Varkoohi et al. [32], and Nasiri Foomani et al. [33]. In contrast, feed conversion traits exhibited lower heritability estimates, which is biologically expected because feed efficiency represents a highly complex phenotype integrating feed intake behavior, digestive efficiency, maintenance energy expenditure, thermoregulation, locomotor activity, and metabolic regulation simultaneously [34,35,36]. Therefore, environmental variation generally contributes more strongly to feed efficiency traits than to body weight traits. Collectively, these findings indicate that growth traits may respond more effectively to direct selection, whereas feed efficiency likely requires multi-trait and physiology-oriented breeding approaches.

The Gompertz growth model showed excellent agreement with individual growth trajectories, with R^2^ values ranging between 0.9892 and 0.9999. Similar results have previously been reported for Japanese quail and other poultry species, where the Gompertz function was identified as one of the most biologically interpretable nonlinear growth models [15,37,38]. The moderate heritability estimates obtained for β_0_, β_1_, β_2_, IPA, and IPW suggest that growth curve characteristics are partially under additive genetic control. In particular, inflection point parameters are biologically important because they reflect maturation timing and maximum growth velocity during the production period [39]. These findings indicate that growth curve parameters may provide additional selection criteria beyond conventional body weight measurements by describing developmental dynamics rather than final growth alone.

Carcass traits exhibited low-to-moderate heritability estimates, with cold carcass and breast weights showing relatively stronger additive genetic determination than abdominal fat and wing traits. Similar genetic parameter estimates for carcass traits have previously been reported in Japanese quail and broiler populations [40,41,42]. Compared with body weight traits, carcass characteristics are generally more sensitive to environmental variation, slaughter conditions, physiological maturity, and nutritional factors, which may partly explain their lower heritability estimates. Nevertheless, the existence of measurable additive genetic variation suggests that economically important carcass characteristics may still respond to selective breeding.

Behavioral traits generally exhibited lower heritability estimates than growth and carcass traits, indicating substantial environmental influence on behavioral expression. Similar findings have frequently been reported in poultry behavioral genetics studies, where behavioral phenotypes are considered highly dynamic and environmentally responsive traits influenced by social interactions, stocking density, stress responsiveness, and housing conditions [6,43]. However, feather pecking, aggressive pecking, and wing stretching exhibited higher heritability estimates than several routine activity behaviors, supporting previous reports indicating that welfare-related behaviors possess measurable additive genetic backgrounds [7,8,9,44]. Particularly, feather pecking behavior has been associated with fear responsiveness, serotonergic activity, social hierarchy dynamics, and stress sensitivity in poultry populations [44]. The genetic variation observed for feather pecking and aggressive pecking may reflect differences among individuals in social reactivity, competitive behavior, and stress responsiveness. These behaviors are not merely isolated social events but may represent broader behavioral mechanisms associated with fearfulness, dominance interactions, and sensitivity to environmental challenges. Therefore, the moderate heritability estimates obtained for feather pecking and aggressive pecking suggest that part of the individual variation in these welfare-relevant behaviors is under additive genetic control. Nevertheless, because these behaviors are also strongly influenced by social environment and group structure, their potential use in breeding strategies should be considered cautiously and validated in larger populations.

The substantially higher phenotypic variability observed for behavioral traits compared with growth and carcass traits further supports the dynamic nature of poultry behavior. Particularly, high coefficients of variation for wing stretching, feather pecking, scratching, and drinking behaviors indicate substantial inter-individual differences in behavioral responsiveness. Similar behavioral variability has previously been reported in both quail and chicken populations and is generally attributed to the strong environmental sensitivity and social plasticity of behavioral phenotypes [45,46,47,48]. Despite this environmental sensitivity, the existence of measurable heritability estimates indicates that behavioral traits still contain exploitable additive genetic variation.

Correlation analyses revealed biologically meaningful relationships between activity-related behaviors and production traits. Feeding behavior showed favorable genetic and phenotypic correlations with BW42, whereas inactivity behavior was positively associated with body weight and carcass traits but negatively associated with FCR42. Similar positive relationships between feeding activity and growth performance have previously been reported in broiler chickens, ducks, and quail populations [49,50,51]. In contrast, walking behavior exhibited negative relationships with BW42, indicating that more active individuals tended to exhibit lower growth performance. Similar reductions in spontaneous locomotor activity have consistently been observed in fast-growing poultry genotypes subjected to intensive selection for rapid growth and feed efficiency [1,2,52,53]. These relationships support the energy allocation hypothesis, which proposes that animals partition limited metabolic energy among growth, maintenance, thermoregulation, and locomotor activity [54,55]. Consequently, increased locomotor activity may reduce the proportion of available energy allocated to tissue accretion and growth processes. The relationships between activity-related behaviors and production traits may also be interpreted within an energy allocation framework. Individuals expressing greater feeding and inactivity tended to show more favorable growth and carcass-related performance, whereas walking behavior was negatively associated with BW42. This pattern suggests that birds with higher locomotor activity may allocate a greater proportion of available energy toward movement and environmental exploration rather than tissue accretion. Conversely, heavier birds may express lower spontaneous activity because of increased body mass and reduced locomotor motivation. Thus, the observed correlations between behavior and production traits likely reflect a biological trade-off between growth, activity, and maintenance processes rather than simple direct causality.

Compared with activity-related behaviors, welfare-related behavioral traits exhibited relatively weak relationships with production characteristics. Most genetic and phenotypic correlations between social behaviors and growth traits were low and statistically non-significant, suggesting that social behaviors may be partially independent from classical production-related physiological pathways. Similar findings have been reported in poultry behavioral genetics studies, where social and welfare-related behaviors were shown to possess distinct genetic architectures from growth and carcass traits [6,9,47]. Nevertheless, strong genetic correlations observed among several social behaviors indicate that these traits are not genetically independent from one another but instead appear to form interconnected behavioral networks associated with social reactivity and stress responsiveness. Similar genetically correlated behavioral syndromes involving fearfulness, aggression, and social responsiveness have previously been described in poultry populations [47,56]. Particularly, the positive relationship between shaking and aggressive pecking behaviors may indicate that individuals exhibiting elevated social reactivity are simultaneously more prone to aggressive interactions. These findings suggest that social behaviors should not be interpreted as isolated phenotypes but rather as components of broader behavioral architectures shaped by common neurobiological and genetic mechanisms.

An additional limitation of the present study concerns the estimation of feed conversion ratio (FCR). Feed intake was recorded at the pen level rather than for individual birds, resulting in identical feed intake information for individuals housed within the same pen. Consequently, the calculated FCR values should be interpreted as individual gain-adjusted feed conversion indicators rather than true individual feed efficiency measurements. This approach may reduce the amount of individual variation captured for feed intake and may partially confound pen-level environmental influences with individual FCR estimates. Therefore, genetic correlations involving FCR should be interpreted cautiously, as their magnitude and precision may differ from those obtained using individually recorded feed intake data.

A limitation of the present study is the relatively moderate pedigree size used for the estimation of genetic parameters. Although the pedigree included 1347 individuals across three generations and allowed reliable estimation of most variance components, some behavioral traits exhibited low heritability estimates accompanied by relatively large standard errors. Consequently, genetic correlations involving these traits may be associated with greater uncertainty than those estimated for growth and carcass traits. Therefore, the reported correlations should be interpreted with caution and validated in larger populations and breeding programs with more extensive pedigree or genomic information. Another limitation of the present study is the absence of genomic information. The genetic parameter estimates were derived from pedigree-based animal models, which may not capture the full extent of Mendelian sampling variation among individuals. The incorporation of genomic data through approaches such as genomic BLUP or single-step genomic evaluation could improve the accuracy of relationship estimates and provide additional insight into the genetic architecture of behavioral and production traits. Future studies integrating pedigree and genomic information may therefore contribute to a more comprehensive understanding of welfare-related traits in poultry populations.

## 5. Conclusions

The present study demonstrated that behavioral traits in Japanese quail possess measurable additive genetic variation and exhibit biologically meaningful relationships with growth and carcass characteristics. Body weight traits showed moderate-to-high heritability estimates, whereas behavioral traits generally exhibited lower but still exploitable levels of genetic variation. Among the evaluated behaviors, feather pecking, aggressive pecking, and wing stretching displayed the highest heritability estimates. Furthermore, feeding, inactivity, and walking behaviors showed notable genetic relationships with production traits, suggesting that behavioral and production phenotypes are partially interconnected through common biological mechanisms. The generally weak correlations observed between most welfare-related behavioral traits and production characteristics indicate that the incorporation of selected behavioral traits into breeding objectives may be feasible without causing major adverse effects on productive performance. These findings provide further evidence that behavioral traits may serve as useful complementary indicators in poultry breeding programs aiming to improve animal welfare alongside conventional production traits.

## Figures and Tables

**Table 1:** Goodness-of-fit statistics for alternative nonlinear growth models fitted to Japanese quail growth data.

Model	AIC	BIC	R^2^
Logistic	16.34	16.18	0.9994
Richards	15.13	14.92	0.9995
MMF	15.92	15.7	0.9992
Gompertz	13.27	13.12	0.9997
von Bertalanffy	16.47	16.32	0.9989

AIC: Akaike Information Criterion; BIC: Bayesian Information Criterion; R^2^: coefficient of determination; MMF: Morgan–Mercer–Flodin model. Lower AIC and BIC values indicate better model fit. The Gompertz model exhibited the best overall fit among the evaluated nonlinear growth functions.

**Table 2:** Descriptive statistics and heritability estimates for growth performance traits in Japanese quail.

Trait	Mean	SD	CV (%)	Min.	Max.	h^2^ ± SE
BW35 (g)	140.00	14.88	10.63	108.00	186.24	0.55 ± 0.01
BW42 (g)	175.74	15.98	9.09	140.80	226.60	0.53 ± 0.01
FCR35	3.05	0.37	12.12	2.22	4.08	0.28 ± 0.01
FCR42	3.36	0.36	10.57	2.53	4.27	0.34 ± 0.02

BW35: body weight at 35 days of age; BW42: body weight at 42 days of age; FCR35: cumulative feed conversion ratio at 35 days of age; FCR42: cumulative feed conversion ratio at 42 days of age. SD: standard deviation; CV (%): coefficient of variation expressed as percentage; Min.: minimum observed value; Max.: maximum observed value; h^2^ ± SE: heritability estimate and its standard error.

**Table 3:** Descriptive statistics and heritability estimates for Gompertz growth curve parameters in Japanese quail.

Trait	Mean	SD	CV (%)	Min.	Max.	h^2^ ± SE
β_0_ (g)	363.01	93.53	25.77	198.89	692.00	0.25 ± 0.01
β_1_	4.78	0.54	11.33	3.43	6.73	0.27 ± 0.02
β_2_	0.05	0.01	18.13	0.03	0.07	0.20 ± 0.02
IPA (day)	33.86	6.28	18.55	21.49	51.84	0.25 ± 0.02
IPW (g)	133.31	33.66	25.25	73.04	233.97	0.25 ± 0.01

β_0_: asymptotic mature weight; β_1_: integration constant; β_2_: growth rate parameter; IPA: age at inflection point; IPW: body weight at inflection point. SD: standard deviation; CV (%): coefficient of variation expressed as percentage; Min.: minimum observed value; Max.: maximum observed value; h^2^ ± SE: heritability estimate and its standard error.

**Table 4:** Descriptive statistics and heritability estimates for slaughter and carcass traits in Japanese quail.

Trait	Mean	SD	CV (%)	Min.	Max.	h^2^ ± SE
Cold carcass (g)	133.88	15.42	11.52	96.11	176.25	0.31 ± 0.04
Breast (g)	46.27	6.14	13.27	29.88	63.17	0.28 ± 0.04
Thigh (g)	31.18	4.92	15.78	20.44	43.81	0.25 ± 0.04
Wing (g)	10.42	1.51	14.49	6.94	14.87	0.18 ± 0.03
Abdominal fat (g)	2.84	0.92	32.39	0.71	5.96	0.12 ± 0.03

SD: standard deviation; CV (%): coefficient of variation expressed as percentage; Min.: minimum observed value; Max.: maximum observed value; h^2^ ± SE: heritability estimate and its standard error.

**Table 5:** Descriptive statistics and heritability estimates for behavioral traits in Japanese quail.

Trait	Mean	SD	CV (%)	Min.	Max.	h^2^ ± SE
Feeding	22.08	27.75	125.66	0.00	103.00	0.12 ± 0.08
Drinking	6.64	8.77	131.99	0.00	50.00	0.16 ± 0.04
Walking	35.17	23.56	66.99	0.00	162.00	0.15 ± 0.02
Inactivity	176.27	45.41	35.14	5.00	277.50	0.14 ± 0.02
Scratching	6.31	9.93	157.40	0.00	68.00	0.12 ± 0.08
Wing stretching	0.62	1.22	196.24	0.00	7.00	0.34 ± 0.021
Shaking	1.79	1.81	100.89	0.00	8.50	0.15 ± 0.04
Preening	33.46	22.66	67.71	0.00	148.50	0.06 ± 0.05
Feather pecking	0.76	1.44	187.76	0.00	8.00	0.35 ± 0.01
Aggressive pecking	2.99	3.24	108.57	0.00	17.50	0.33 ± 0.02

SD: standard deviation; CV (%): coefficient of variation expressed as percentage; Min.: minimum observed value; Max.: maximum observed value; h^2^ ± SE: heritability estimate and its standard error.

**Table 6:** Genetic (r_G_) and phenotypic (r_P_) correlations between performance-related activity behaviors and production traits in Japanese quail.

Trait	BW42	FCR42	IPA	Cold Carcass
Feeding	r_G_ = 0.45 (0.01)	r_G_ = −0.15 (0.03)	r_G_ = −0.29 (0.03)	r_G_ = 0.03 (0.06)
	r_P_ = 0.36 (0.000 *)	r_P_ = −0.10 (0.041 *)	r_P_ = −0.25 (0.000 *)	r_P_ = −0.02 (0.752)
Drinking	r_G_ = 0.07 (0.06)	r_G_ = −0.01 (0.08)	r_G_ = 0.05 (0.09)	r_G_ = 0.02 (0.07)
	r_P_ = 0.04 (0.383)	r_P_ = −0.00 (0.981)	r_P_ = 0.04 (0.395)	r_P_ = −0.02 (0.736)
Walking	r_G_ = −0.38 (0.02)	r_G_ = 0.01 (0.06)	r_G_ = 0.10 (0.05)	r_G_ = 0.08 (0.03)
	r_P_ = −0.33 (0.000 *)	r_P_ = 0.11 (0.792)	r_P_ = 0.09 (0.072)	r_P_ = 0.03 (0.555)
Inactivity	r_G_ = 0.42 (0.01)	r_G_ = −0.26 (0.06)	r_G_ = −0.12 (0.06)	r_G_ = 0.38 (0.01)
	r_P_ = 0.34 (0.000 *)	r_P_ = −0.22 (0.000 *)	r_P_ = −0.10 (0.594)	r_P_ = 0.32 (0.000 *)
Scratching	r_G_ = 0.07 (0.06)	r_G_ = 0.01 (0.08)	r_G_ = 0.05 (0.09)	r_G_ = 0.02 (0.07)
	r_P_ = 0.04 (0.381)	r_P_ = 0.00 (0.982)	r_P_ = 0.04 (0.394)	r_P_ = −0.02 (0.737)

r_G_: genetic correlation; r_P_: phenotypic correlation; values in parentheses for r_G_ indicate standard errors (SE), whereas values in parentheses for r_P_ indicate probability levels (*p*-values); BW42: body weight at 42 days of age; FCR42: cumulative feed conversion ratio at 42 days of age; IPA: age at inflection point; * *p* < 0.05.

**Table 7:** Genetic (r_G_) and phenotypic (r_P_) correlations between welfare-related behavioral traits and production traits in Japanese quail.

Trait	BW42	FCR42	IPA	Cold Carcass
Wing stretching	r_G_ = 0.11 (0.06)	r_G_ = −0.08 (0.06)	r_G_ = −0.02 (0.10)	r_G_ = 0.01 (0.11)
	r_P_ = 0.09 (0.082)	r_P_ = −0.06 (0.218)	r_P_ = −0.01 (0.834)	r_P_ = 0.01 (0.888)
Shaking	r_G_ = −0.10 (0.06)	r_G_ = 0.10 (0.07)	r_G_ = −0.11 (0.05)	r_G_ = −0.09 (0.07)
	r_P_ = −0.10 (0.055)	r_P_ = 0.09 (0.072)	r_P_ = −0.09 (0.075)	r_P_ = −0.08 (0.119)
Preening	r_G_ = 0.04 (0.07)	r_G_ = 0.02 (0.11)	r_G_ = −0.09 (0.06)	r_G_ = 0.07 (0.08)
	r_P_ = −0.04 (0.484)	r_P_ = 0.02 (0.712)	r_P_ = −0.08 (0.145)	r_P_ = 0.06 (0.289)
Aggressive pecking	r_G_ = 0.01 (0.08)	r_G_ = 0.07 (0.07)	r_G_ = −0.07 (0.07)	r_G_ = −0.01 (0.09)
	r_P_ = 0.01 (0.910)	r_P_ = 0.07 (0.182)	r_P_ = −0.06 (0.255)	r_P_ = −0.01 (0.889)

r_G_: genetic correlation; r_P_: phenotypic correlation; values in parentheses for r_G_ indicate standard errors (SE), whereas values in parentheses for r_P_ indicate probability levels (*p*-values). BW42: body weight at 42 days of age; FCR42: cumulative feed conversion ratio at 42 days of age; IPA: age at inflection point. Welfare-related behavioral traits included wing stretching, shaking, preening, and aggressive pecking.

## Data Availability

The data that support the findings of this study are available from the corresponding author upon reasonable request.

## Supplementary Materials

The following supporting information can be downloaded at: https://www.mdpi.com/article/10.3390/ani16131943/s1, Table S1. Sex effects for growth, growth curve, carcass, and behavioral traits in Japanese quail.
