# Supplementary material for: Heritability Estimates of Behavioral Traits and Their Genetic Relationships with Performance Traits in Japanese Quail
Source: Animals (Basel). 2026 Jun 23;16(13):1943. doi: 10.3390/ani16131943 (PMC13360013; doi:10.3390/ani16131943)
Supplement: Supplementary file 1 [file animals-16-01943-s001.zip › animals-4360725-supplementary.pdf]

Supplementary Table S1. Sex effects for growth, growth curve, carcass, and behavioral traits in Japanese quail.

Trait	Female (Mean ± SD)	Male (Mean ± SD)	P-value
BW35	143.98 ± 15.18	135.33 ± 13.28	<0.001
BW42	181.99 ± 15.84	168.51 ± 13.05	<0.001
FCR35	2.97 ± 0.37	3.15 ± 0.36	<0.001
FCR42	3.25 ± 0.34	3.50 ± 0.33	<0.001
β_0	380.56 ± 92.02	343.39 ± 94.11	<0.001
β_1	4.91 ± 1.12	4.70 ± 0.56	0.020
β_2	0.05 ± 0.01	0.05 ± 0.01	0.647
IPA	34.38 ± 6.12	33.34 ± 6.68	0.109
IPW	140.01 ± 33.86	126.34 ± 34.63	<0.001
Cold Carcass	122.70 ± 11.00	115.15 ± 9.93	<0.001
Breast	47.20 ± 5.28	44.66 ± 5.17	<0.001
Thigh	28.11 ± 3.11	26.25 ± 2.95	<0.001
Wing	10.55 ± 1.48	10.00 ± 1.37	<0.001
Back-neck	35.87 ± 5.54	33.39 ± 5.19	<0.001
Feeding	21.4 ± 29.1	22.9 ± 26.2	0.536
Drinking	6.5 ± 9.1	6.8 ± 8.4	0.721
Walking	33.6 ± 24.5	36.9 ± 22.4	0.108
Inactivity	179.8 ± 46.8	172.2 ± 43.9	0.067
Scratching	6.4 ± 10.2	6.2 ± 9.5	0.842
Wing stretching	0.56 ± 1.23	0.69 ± 1.20	0.284
Shaking	1.73 ± 1.87	1.86 ± 1.74	0.463
Preening	35.1 ± 23.7	31.6 ± 21.4	0.093
Feather pecking	0.67 ± 1.39	0.86 ± 1.48	0.171
Aggressive pecking	2.54 ± 3.12	3.51 ± 3.29	0.058

Values are presented as mean ± SD for females and males. P-values represent the fixed effect of sex from the corresponding model used in the study. BW35 and BW42: body weight at 35 and 42 days of age; FCR35 and FCR42: cumulative feed conversion ratio at 35 and 42 days of age; β0, β1, and β2: Gompertz growth parameters; IPA: age at inflection point; IPW: body weight at inflection point.
